# Investigating causality in associations between smoking initiation and schizophrenia using Mendelian randomization

**DOI:** 10.1038/srep40653

**Published:** 2017-01-19

**Authors:** Suzanne H. Gage, Hannah J. Jones, Amy E. Taylor, Stephen Burgess, Stanley Zammit, Marcus R. Munafò

**Affiliations:** 1 MRC Integrative Epidemiology Unit (IEU) at the University of Bristol, Bristol, UK; 2 UK Centre for Tobacco and Alcohol Studies, School of Experimental Psychology, University of Bristol, Bristol, UK; 3 School of Social and Community Medicine, University of Bristol, Bristol, UK; 4 Department of Public Health and Primary Care, University of Cambridge, Cambridge, UK; 5 MRC Centre for Neuropsychiatric Genetics and Genomics, Cardiff University, Cardiff, UK

## Abstract

Smoking is strongly associated with schizophrenia. Although it has been widely assumed that this reflects self-medication, recent studies suggest that smoking may be a risk factor for schizophrenia. We performed two-sample bi-directional Mendelian randomization using summary level genomewide association data from the Tobacco And Genetics Consortium and Psychiatric Genomics Consortium. Variants associated with smoking initiation and schizophrenia were combined using an inverse-variance weighted fixed-effects approach. We found evidence consistent with a causal effect of smoking initiation on schizophrenia risk (OR 1.73, 95% CI 1.30–2.25, p < 0.001). However, after relaxing the p-value threshold to include variants from more than one gene and minimize the potential impact of pleiotropy, the association was attenuated (OR 1.03, 95% CI 0.97–1.09, p = 0.32). There was little evidence in support of a causal effect of schizophrenia on smoking initiation (OR 1.01, 95% CI 0.98–1.04, p = 0.32). MR Egger regression sensitivity analysis indicated no evidence for pleiotropy in the effect of schizophrenia on smoking initiation (intercept OR 1.01, 95% CI 0.99–1.02, p = 0.49). Our findings provide little evidence of a causal association between smoking initiation and schizophrenia, in either direction. However, we cannot rule out a causal effect of smoking on schizophrenia related to heavier, lifetime exposure, rather than initiation.

It has long been known that smoking is harmful to physical health, but less is known about the nature of its association with mental health. Smoking rates are markedly higher among individuals with mental health disorders than in the general population, and while smoking rates have fallen over recent decades, corresponding decreases have not been seen in mental health populations^1^. In particular, smoking is strongly associated with psychotic disorders such as schizophrenia^2^. Historically, up to around 80% of patients with schizophrenia were smokers^3^, and more recent estimates suggest that the prevalence of smoking in inpatients is around 60%^4^,^5^. There is evidence that supports a self-medication model to explain this association: constituents of tobacco smoke can increase the metabolism of anti-psychotic drugs^6^, and there is also some evidence that nicotine might reduce some of the cognitive impairment side effects of drugs such as haloperidol^7^. Nicotine has also been implicated in improving various physiological deficits associated with schizophrenia, including pre-pulse inhibition^8^ and the P50 wave^9^.

Recently, however, some evidence has emerged to suggest that the association between smoking and schizophrenia could be operating in the opposite direction. A systematic review and meta-analysis of cross sectional and prospective studies of smoking and psychotic outcomes indicated that daily tobacco use was associated with both an increased risk of psychosis, and earlier age of onset of a disorder^10^ – although this study did not address confounding presenting only unadjusted estimates. A recent large prospective study of Swedish registry data similarly found that light and heavy cigarette smoking was associated with incident risk of schizophrenia^11^. The association was dose-dependent, and persisted (though was attenuated) after adjustment for socioeconomic status, drug use and degree of familial genetic overlap. Perhaps most interestingly, in a recent genomewide association study (GWAS) of schizophrenia^12^, a variant (rs8042374) within the *CHRNA5-A3-B4* gene cluster strongly associated with heaviness of smoking in smokers (p = 2.4 × 10^−24^ in the cigarettes-per-day GWAS^13^) was one of the variants to reach genomewide significance. This could mean that there is shared genetic architecture between schizophrenia and smoking, but it could also indicate a causal association between smoking and schizophrenia^14^,^15^. It is plausible that the association could be causal in either or both directions, but this is very hard to establish using standard observational epidemiological methods. For example, an earlier Swedish longitudinal study found evidence of an association in the opposite direction to the study discussed above^16^.

One approach to investigate causality in observational studies is Mendelian randomization (MR), whereby genetic variants that predict an observational exposure are used as unconfounded proxy measures for the exposure itself. Associations between the variants and the outcome of interest can thus provide evidence of causation whilst, subject to certain assumptions, eliminating problems of confounding and reverse causation. A recent Mendelian randomization study found an association between a SNP in the nicotinic acetylcholine receptor alpha 3 subunit (*CHRNA3*) gene strongly associated with heaviness of smoking (rs1051730) and likelihood of being prescribed anti-psychotic medication, which was used as a proxy for risk of schizophrenia, in a sample of smokers^17^.

Since genetic variants typically explain a small proportion of the variance in the exposure, very large sample sizes are needed, which can make conducting such studies challenging. Recently, the two-sample MR method has been developed, whereby data on gene-exposure and gene-outcome associations from different samples can be used to conduct MR analyses^18^. This means that publicly-available summary data from genomewide association studies can be used to assess causal associations between exposures and outcomes, increasing sample size, and therefore power to detect causal associations.

We used two-sample MR to assess the causal effect of smoking initiation on schizophrenia (using genetic variants associated with smoking initiation), and of schizophrenia risk on smoking initiation, cigarettes per day, and cessation (using genetic variants associated with schizophrenia risk), in an attempt to clarify the causal relationship between smoking and schizophrenia.

## Results

### Schizophrenia risk and smoking initiation

**Figure 1:**
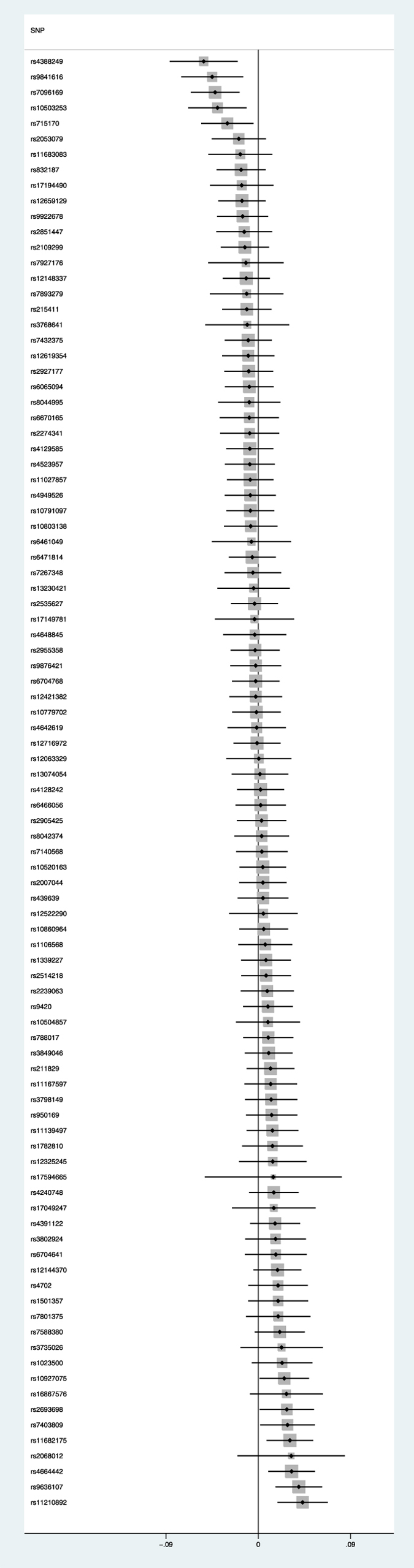
Forest plot showing association with smoking initiation (odds ratio and 95% confidence interval) for each genomewide-significant schizophrenia SNP.

The 94 SNPs used as a proxy for risk of schizophrenia case status provided little evidence of a causal effect of schizophrenia risk on likelihood of being a smoker (OR per 1 unit increase in log odds of schizophrenia 1.02, (95% CI 0.98–1.06, p = 0.32). This translates to an odds ratio of 1.01 (95% CI 0.98–1.04) for smoking per doubling of odds of schizophrenia (see Fig. 1). There was moderate heterogeneity (I^2^  =  38%, 95% CI 20–52% p = 0.0002), but the random-effects estimate was very similar to the fixed effects model (OR 1.01, 95% CI 0.98–1.05, p = 0.37). When we performed MR Egger regression, there was no evidence that the intercept (representing bias in the causal estimate due to pleiotropy) differed from the null (intercept OR 1.01, 95% CI 0.99–1.02, p = 0.49). The slope point estimate (representing the causal effect estimate of schizophrenia on smoking initiation) did not statistically differ from the null (OR 0.95, 95% CI 0.78–1.16, p = 0.61).

### Schizophrenia risk and other smoking phenotypes

The 94 SNPs used as a proxy for risk of schizophrenia case status provided little evidence of a causal effect of schizophrenia risk on smoking heaviness in 38,181 daily smokers in the TAG consortium (OR per unit increase in log odds of schizophrenia 1.03; 95% CI 0.86–1.24, p = 0.76). There was also little evidence of a causal effect of schizophrenia risk on odds of smoking cessation among 35,845 smokers in the TAG consortium (OR 0.97; 95% CI 0.93–1.03, p = 0.22).

### Smoking initiation and schizophrenia risk

The 4 SNPs used as a proxy for likelihood of being an ever smoker provided some evidence of a causal effect of smoking status on risk of schizophrenia (OR per 1 unit increase in log odds of being a smoker 2.17; 95% CI 1.46–3.23, p < 0.001). This equates to an odds ratio of 1.71 (95% CI 1.30–2.25) of schizophrenia per doubling in odds of smoking. When only the most strongly associated SNP (rs6265) was analysed, the association was similar (OR 1.98; 95% CI 1.03–3.58) Due to the correlated nature of the SNPs, it was not possible to perform MR Egger regression as a sensitivity analysis to test the assumption of no biological pleiotropy.

Using genetic variants associated with smoking initiation at a less stringent significance threshold, 21 additional independent SNPs associated with smoking initiation (at p < 10^−6^) provided no evidence of a causal effect of smoking status on risk of schizophrenia (OR per doubling in odds of being a smoker including one BDNF SNP (rs6265) OR 1.03; 95% CI 0.97–1.09, p = 0.32). Heterogeneity in this analysis was somewhat large, (I^2^ = 61.3, 95% CI 38–76%, p < 0.001). However, MR Egger regression suggested no evidence of pleiotropy (intercept OR 1.03, 95% CI 1.00–1.06, p = 0.061), and weak evidence of causality (slope OR 0.69, 95% CI 0.43–1.08, p = 0.118). Results were similar if we excluded rs6265.

## Discussion

### Schizophrenia risk and smoking initiation

Our results did not support a possible causal effect of schizophrenia risk on smoking initiation. Sensitivity analyses suggested that although there was some heterogeneity in the results, there was no clear evidence for biological pleiotropy impacting on the findings, meaning that bias due to violation of one of the key assumptions of MR (i.e., that any genetic effect on the outcome only occurs via the exposure, and not directly) was unlikely to be substantial. The MR Egger regression causal estimate did not differ from the null. Although the point estimate was in the opposite direction, the confidence intervals were consistent with our main findings.

### Smoking initiation and schizophrenia risk

When using only genomewide significant SNPs, our results were consistent with a possible causal effect of smoking initiation on schizophrenia risk. However, this result was somewhat hard to interpret as only four SNPs were used in this risk score, and all of these were from the same gene region (*BDNF*). These SNPs are all highly correlated, and although correlation was taken in to account in the MR model, the risk of biological pleiotropy (i.e., an effect of gene on the outcome not via smoking) was much harder to rule out; *BDNF* and its associated SNPs have reached genomewide significance in a number of other GWAS, including those of obesity and body mass index^19^,^20^, and caffeine consumption^21^.

Although no SNPs in *BDNF* reach genomewide significance in the schizophrenia GWAS, it is a gene that has been implicated in schizophrenia previously, particularly in relation to cognitive deficits associated with the disease^22^, as well as other psychiatric disorders^23^. Some studies have suggested that smoking might be a method by which people with schizophrenia alleviate cognitive difficulties, although other studies suggest cognition is worse in people with schizophrenia who smoke^24^. Furthermore, given the higher prevalence of smoking in populations with mental health problems, it is possible that the smoking initiation GWAS sample might be enriched for mental health problems compared to the general population, meaning that variants identified could be causally linked to these mental health problems rather than smoking. This result must therefore be interpreted with particular caution; the association between SNPs in the *BDNF* gene area and schizophrenia *may* reflect a causal association between smoking initiation and schizophrenia, but could also potentially be a pleiotropic effect, either via one of the already identified alternative pathways such as obesity or caffeine, or via another currently unidentified pathway. It was not possible to conduct an MR Egger regression using these 4 SNPs to test whether these smoking initiation SNPs were causally related to schizophrenia due to the high correlation between the smoking SNPs, and the small number of them.

Our analysis using a lower p-value cutoff found no evidence for an association between smoking initiation and risk of schizophrenia, and this also supports the interpretation that this was a pleiotropic effect, although in 2-sample MR, the use of weaker instruments such as these can result in bias towards the null.

### Smoking heaviness phenotype

We did not use cigarettes per day or smoking cessation SNPs as exposure variables, as we could not stratify these data by smoking status. The SNP that strongly predicts cigarettes per day (rs1051730) does not predict smoking initiation, and only exerts an effect on smoking heaviness after a person has become an established smoker^25^. The GWAS that identified it was conducted in a sample of daily smokers, unlike that for smoking initiation which was a sample that included ever smokers and never smokers^13^. This genetic variant has been shown to be associated with schizophrenia risk as a highly correlated SNP (rs8042374) reached genomewide significance in the PGC2 GWAS of schizophrenia^12^ (used in these analyses), suggesting a possible causal role of smoking heaviness on schizophrenia. However, as the analysis in the current study is based on summary data, we cannot stratify the sample to investigate whether this association is seen in non-smokers (indicating shared genetic aetiology of the two traits) or only in regular smokers (indicating a direct causal association). Whilst this SNP has been identified in unstratified GWAS of outcomes where a causal association with smoking is known (e.g., lung cancer), this has only been the case where effect sizes are sufficiently large that the association can survive this dilution. In order to glean more information about causality (versus direct biological pleiotropy, where the SNP in question has an effect on both smoking and schizophrenia), a stratified analysis would be required, to check for a lack of association in non-smokers. To perform a 2-sample analysis on the unstratified PGC2 GWAS would merely repeat what is already known from the GWAS, that this SNP is associated with schizophrenia risk, but would not help us understand causality.

### Strengths and limitations

One strength of two-sample MR is that it offers very large sample sizes for analyses. Our analyses in both directions are based on such samples, which should provide sufficient power to identify small effects that are likely in the context of complex phenotypes such as schizophrenia risk and smoking initiation. However, even in these designs we are still underpowered to detect extremely small effect sizes given the small amount of variance explained by the SNPs we have used. For example, rs6265 explains 0.03% of the variance in smoking initiation, meaning we have approximately 86% power to detect an effect size of OR 1.1 of smoking initiation on schizophrenia risk.

It is important to note that the genetic risk for schizophrenia is based on a case-control GWAS, and therefore this might affect our interpretation of the association between schizophrenia and smoking. Schizophrenia is a rare outcome, and most of the TAG sample will not have schizophrenia. Therefore, we are making an assumption here that genetic risk for schizophrenia might be inducing subclinical schizophrenia symptoms in those that do not reach clinical diagnosis. There is some evidence that this is the case^26^, though it is not yet strong or consistent^27^.

A further potential limitation could be the role of maternal smoking, as this may be a genetic confounder, influencing both the genotype of the individual, and the relationship between smoking and schizophrenia in the individual. However, the impact of maternal smoking would need to be substantial in order to affect this analysis, given that smoking initiation is independent from smoking during pregnancy and therefore likely influenced by different genetic variants^13^, and offspring share on average 50% of their genotype with their mothers.

One final potential limitation of this study is the possibility of population stratification due to the different ancestry samples included in the PGC2 GWAS (both European and Asian). There is some evidence of a slight difference in allele frequency for rs6265 between European and Asian populations (A allele frequency 0.2 in Europeans and 0.4 in Asians according to hapmap)^28^. The PGC2 GWAS adjusted for principal components, which should mitigate the effect of population stratification somewhat.

The association between smoking, schizophrenia and other childhood mental health is likely to be complex, in terms of both environmental and genetic risk factors. Childhood mental health predicts both smoking and risk of schizophrenia. Therefore the impact of different forms of pleiotropy on the associations between schizophrenia and smoking is important to consider. While biological pleiotropy (where one variant has multiple independent effects on different phenotypes) violates the assumptions of MR, mediated pleiotropy (where one variant influences a phenotype via intermediate mechanisms upstream of that phenotype) is not so problematic. For example, smoking could influence adolescent mental health, which could in turn increase the risk of more serious mental health problems such as schizophrenia. This would still be consistent with a causal effect of smoking on schizophrenia. We conducted MR Egger regressions to formally test for biological pleiotropy, and found little evidence for this in either direction.

### Comparison to previous literature

Although the evidence from this study for smoking initiation as a risk factor for schizophrenia is weak, other studies posit this as a plausible explanation for high rates of smoking seen in individuals with schizophrenia. Given that there are very few modifiable risk factors presently identified for this hugely debilitating disease further investigation of this question is therefore warranted. Stronger evidence to support or refute a causal effect of smoking might also inform our understanding of the relationship between cannabis use and schizophrenia, given the common co-occurrence of cannabis and tobacco use^29^; however, it seems highly unlikely that smoking could explain this association given that psychotic experiences are often described during acute intoxication with cannabis, but not with tobacco.

In order to determine whether the association between smoking and schizophrenia is causal, conventional MR using SNPs associated with heaviness of smoking would be informative, if a large sample could be identified with information on both schizophrenia case status and smoking status. One study has attempted this using anti-psychotic medication as a proxy for schizophrenia^17^. They found weak evidence using MR that higher cigarette consumption amongst smokers was associated with increased likelihood of being on antipsychotic medication, although it is not possible to rule out a direct effect of the SNP (rs1051730) on the outcome from these data. In that study, the OR was substantially smaller in their sample of non-smokers, although confidence intervals overlapped substantially between the two analyses. It is also important to note that many individuals who are prescribed anti-psychotic medication do not have schizophrenia or a psychotic illness, and therefore this may not be an ideal proxy measure for schizophrenia^30^.

We found little evidence that schizophrenia risk was associated with uptake of smoking. This is in contrast to previous suggestions that the association between smoking and schizophrenia could be due to self-medication with nicotine. Evidence from observational epidemiology is broadly consistent with our present findings, namely that smoking initiation appears to predate psychotic experiences or schizophrenia^31^. Our findings are also consistent with a study that used cross-trait LD-score regression to examine the genetic correlation between smoking initiation and schizophrenia^32^. This study found no evidence of a genetic correlation, although it did find weak evidence of associations between schizophrenia and age of smoking initiation, and cigarettes per day. MR analyses are more suited to assessing causality than LD-score regression (which targets genetic correlation). MR uses variants identified as being good proxies for modifiable risk factors to provide a directional estimate of the association between the risk factor and the outcome. In contrast, LD-score analysis uses variants across the whole genome and is a symmetric (i.e., non-directional) analysis of the risk factor and the outcome, meaning that LD-score regression is not able to ascertain the direction of causation between the risk factor and outcome. Also, the contribution of a variant to a MR analysis is proportional to its association with the exposure, whereas the contribution of a variant to LD-score regression is proportional to its “LD-score”. This means that even if a MR investigation was undertaken using genetic variants from the whole genome, it would differ from LD-score regression. The LD-score does not differ between exposures, meaning there is the potential for systematic bias in LD-score regressions across different phenotypes. Finally, the LD-score is likely to be a weak instrument, meaning a higher possibility of bias.

In conclusion, we found little evidence to suggest that schizophrenia risk increases the likelihood of smoking initiation. Our results provide some evidence consistent with a causal effect of smoking on schizophrenia, although a sensitivity analysis where we relaxed the p-value threshold suggested this association could be due to pleiotropy, i.e. that smoking initiation and schizophrenia risk share genetic aetiology, rather than being causally related. Nevertheless, while the current evidence that smoking might be a risk factor for schizophrenia is not compelling, the potential that smoking could be a modifiable risk factor for schizophrenia is a strong driver for further investigation of this question.

## Methods

Single nucleotide polymorphisms (SNPs) associated with smoking initiation, and schizophrenia risk, were identified from genomewide significant hits in the Tobacco And Genetics (TAG) consortium^13^ and the Psychiatric Genetics Consortium (PGC2)^12^ GWAS, respectively.

### SNPs associated with schizophrenia

A GWAS meta-analysis conducted by the PGC identified 128 independent SNPs (in 108 physically distinct loci) that met genomewide significance (p < 5 × 10^−8^) for schizophrenia risk in a sample of 36,989 schizophrenia cases and 113,075 controls. These SNPs explained approximately 3.4% of the variance in schizophrenia risk. There were 51 SNPs from PGC2 that were genotyped in TAG. We used SNAP (https://www.broadinstitute.org/mpg/snap/) and the PhenoScanner tool^33^ to identify proxies in high linkage disequilibrium (r^2^ > 0.9), and were able to find an additional 43 proxy SNPs that were in TAG (see Supplementary Table 1), so that the total number of SNPs for our analysis was 94 (out of a possible 108).

### SNPs associated with smoking initiation

The TAG conducted a GWAS of smoking behaviour on 74,053 individuals, and a replication sample of over 140,000 individuals. A binary ever/never measure of smoking initiation was used as our exposure of interest as opposed to smoking heaviness, as smoking heaviness requires knowledge of participants’ smoking status, which was not available in the PGC2 summary data. Eight SNPs met genomewide significance (p < 5 × 10^−8^) for smoking initiation, all located in the *BDNF* gene region. A number of these SNPs were in very high linkage disequilibrium with each other. Using r^2^ values obtained from SNAP we identified all pairs of SNPs correlated at ≥0.9 and randomly selected one from each pair to keep, dropping the others from the score in a stepwise manner. Very highly correlated SNPs do not give extra information, but can make the model unstable. The remaining four SNPs (see Table 1) were also correlated, so a correlation matrix was created (see Supplementary Table 2) which required modification of the likelihood-based method for estimation of a causal effect (see below)^18^. Given the correlation between these SNPs, we conducted two sensitivity analyses around this question. Firstly, we ran the analysis with only the most strongly associated SNP (rs6265). Secondly, we identified a further 21 independent SNPs (see Supplementary Table 3) associated with smoking initiation at a slightly lower threshold (p < 10^−6^). A correlation matrix was not required for this analysis, as all SNPs were independent of each other. Although relaxing the p-value threshold may introduce more weak instruments in to our analysis, the inclusion of several independent SNPs means pleiotropy is potentially less likely, as in order to pleiotropy to impact, all pleiotropic effects would have to operate in the same direction, which is implausible. It also allows us to be able to undertake MR Egger regression as a sensitivity analysis to more formally assess the impact of biological pleiotropy on the association.

### Statistical analysis

#### Schizophrenia risk on smoking initiation, cigarettes per day, and smoking cessation

Log odds ratios and standard errors for the SNPs associated with schizophrenia were recorded. These SNPs were then identified in the full genomewide results from the TAG consortium for each phenotype, and beta-coefficients and standard errors recorded. The SNP-exposure and SNP-outcome associations for the 94 SNPs present in the PGC2 and TAG GWAS were combined in a fixed effects meta-analysis using an inverse-variance weighted approach. This is equivalent to a weighted regression of the SNP-outcome coefficients on the SNP-exposure coefficients. The results of this analysis were converted to odds ratios, and the resulting output is the odds ratio for smoking initiation, cigarettes per day, and cessation (as appropriate) per unit increase in the log odds ratio of schizophrenia risk. This is somewhat hard to interpret, so ORs were converted (by multiplying by 0.693) in order to represent the odds ratio per doubling in odds of the binary exposure.

#### Smoking initiation on schizophrenia risk

Beta coefficients and standard errors for the four remaining SNPs associated with smoking initiation were recorded from the TAG dataset. These SNPs were identified in the full genomewide results from the PGC2, and log odds ratios and standard errors recorded. The SNP-exposure and SNP-outcome associations were combined using the same method as above, but further developed to allow for consideration of the correlational structure between the SNPs. Again the output was converted to odds ratio, and can be interpreted as the effect on schizophrenia risk per unit increase in the log odds ratio of smoking initiation, then converted to represent an odds ratio for schizophrenia risk per doubling in odds to smoking initiation.

#### Sensitivity analyses

In order to formally test for potential violations of Mendelian randomization assumptions, a number of sensitivity analyses were undertaken. I^2^ statistics were calculated to estimate heterogeneity. We also ran a random-effects model to account for heterogeneity, and performed MR Egger regression^34^. This latter method relaxes the assumption made in MR that the effects of genetic variants on the outcome are entirely mediated via the exposure. This is achieved by allowing an intercept term in the weighted regression of the SNP-outcome coefficients on the SNP-exposure coefficients. The intercept parameter represents the average pleiotropic effect of a SNP on the outcome (the direct effect on the outcome not via the exposure of interest). This intercept value can therefore provide a test of directional pleiotropy; if the intercept term is close to the null, then bias in the causal estimate due to pleiotropy is less likely. The beta coefficient from this regression provides a consistent estimate of the causal effect under the assumption that the pleiotropic effects on SNPs on the outcome are uncorrelated with the associations of the SNPs with the exposure. Due to the small number of SNPs, and the high correlation between them, MR Egger regression could not be performed for the association of smoking initiation on schizophrenia using the SNPs associated at a genomewide level, so we used the 21 additional SNPs at the less stringent p-value threshold for this analysis.

#### Sample overlap

Two-sample Mendelian randomization assumes that there is no overlap of participants between the two data sources used. Both TAG and PGC2 used MIGen data, making up 3.6% of the TAG consortium, and 1.4% of the PGC2 sample. This is not substantial, and any bias from this is likely to be in the direction of the null^35^.

## Additional Information

**How to cite this article**: Gage, S. H. *et al*. Investigating causality in associations between smoking initiation and schizophrenia using Mendelian randomization. *Sci. Rep.* **7**, 40653; doi: 10.1038/srep40653 (2017).

**Publisher's note:** Springer Nature remains neutral with regard to jurisdictional claims in published maps and institutional affiliations.

## Supplementary Material

Supplementary Materials

## Figures and Tables

**Table 1:** List of the SNPs associated with smoking initiation used in the 2-sample analysis, and their associations with smoking initiation, and with schizophrenia.

SNP	Chromosome	Position	Gene region	Reference allele	Ref allele frequency	Smoking Initiation			Schizophrenia		
						Beta	SE	p	Beta	SE	p
rs6265	11	27636492	BDNF	T	0.21	−0.061	0.011	1.8 × 10^−8^	−0.052	0.013	8 × 10^−5^
rs4923460	11	27613365	BDNF	T	0.23	−0.058	0.011	4.1 × 10^−8^	−0.045	0.013	4.2 × 10^−4^
rs1304100	11	27528179	BDNF	A	0.74	0.055	0.01	4.4 × 10^−8^	0.038	0.013	2.7 × 10^−3^
rs6484320	11	27659764	BDNF	A	0.76	0.057	0.01	4.9 × 10^−8^	0.043	0.013	7.5 × 10^−4^
