# Supplementary material for: Investigating causality in associations between smoking initiation and schizophrenia using Mendelian randomization
Source: Sci Rep. 2017 Jan 19;7:40653. doi: 10.1038/srep40653 (PMC5244403; doi:10.1038/srep40653)
Supplement: Supplementary Materials [file srep40653-s1.doc]

**Investigating causality in associations between smoking initiation and schizophrenia using Mendelian randomization: Supplementary Material**

Suzanne H. Gage* ^1,2^, Hannah J. Jones ^1,3^, Amy E. Taylor ^1,2^, Stephen Burgess ^1,4^, Stanley Zammit ^3,5^, Marcus R. Munafò ^1,2^

1. MRC Integrative Epidemiology Unit (IEU) at the University of Bristol, UK
2. UK Centre for Tobacco and Alcohol Studies, School of Experimental Psychology, University of Bristol, UK
3. School of Social and Community Medicine, University of Bristol, UK.
4. Department of Public Health and Primary Care, University of Cambridge, UK.
5. MRC Centre for Neuropsychiatric Genetics and Genomics, Cardiff University, UK

Corresponding author: Suzanne H. Gage, School of Experimental Psychology, University of Bristol, 12a Priory Road, Bristol, BS8 1TU, United Kingdom. E: [suzi.gage@bristol.ac.uk](mailto:suzi.gage@bristol.ac.uk)

Key words: smoking, schizophrenia, genetics, Mendelian randomization

Short title: Mendelian randomisation of smoking and schizophrenia

**Table S1.** List of SNPs genomewide associated with schizophrenia, and their association with smoking initiation. Proxies and their correlation are listed for SNPs not available in TAG consortium.

| SNP | Original SNP if proxy used in analysis (correlation) | Chrom-osome | Nearest gene (within 100k bp) | Reference allele | Ref allele frequency | Gene-schizophrenia estimate (se) | p-value | Gene-smoking initiation estimate (se) | p-value |
| --- | --- | --- | --- | --- | --- | --- | --- | --- | --- |
| rs1023500 |  | 22 | CENPM | T | 0.14 | 0.07 (0.01) | 5.0x10^-8^ | 0.02 (0.02) | 0.122 |
| rs10503253 |  | 8 | CSMD1 | A | 0.22 | 0.07 (0.01) | 2.7x10^-8^ | -0.04 (0.01) | 0.006 |
| rs10520163 |  | 4 | CLCN3 | T | 0.50 | 0.06 (0.01) | 1.0x10^-8^ | 0.00 (0.01) | 0.696 |
| rs10791097 |  | 11 | LOC100507431 | T | 0.47 | 0.07 (0.01) | 2.9x10^-12^ | -0.01 (0.01) | 0.524 |
| rs10803138 |  | 1 | SDCCAG8 | G | 0.73 | 0.07 (0.01) | 1.8x10^-8^ | -0.01 (0.01) | 0.584 |
| rs10860964 |  | 12 | C12orf42 | T | 0.61 | 0.06 (0.01) | 9.9x10^-8^ | 0.01 (0.01) | 0.651 |
| rs11027857 |  | 11 | LOC105376595 | A | 0.52 | 0.06 (0.01) | 3.2x10^-9^ | -0.01 (0.01) | 0.501 |
| rs1106568 |  | 4 | GPM6A | G | 0.26 | 0.07 (0.01) | 1.1x10^-8^ | 0.01 (0.01) | 0.606 |
| rs11139497 |  | 9 | LOC105376107 | A | 0.32 | 0.07 (0.01) | 3.1x10^-9^ | 0.01 (0.01) | 0.283 |
| rs11210892 |  | 1 | PTPRF | G | 0.34 | 0.07 (0.01) | 5.0x10^-10^ | 0.04 (0.01) | 5.4x10^-4^ |
| rs11682175 |  | 2 | LOC105377632 | C | 0.48 | 0.07 (0.01) | 2.5x10^-12^ | 0.03 (0.01) | 0.008 |
| rs12144370 | rs12129573 (0.97) | 1 | LOC105378800 | T | 0.40 | 0.07 (0.01) | 1.2x10^-9^ | 0.02 (0.01) | 0.114 |
| rs12148337 |  | 15 | LOC105370878 | T | 0.46 | 0.06 (0.01) | 5.3x10^-8^ | -0.01 (0.01) | 0.320 |
| rs12325245 |  | 16 | CNOT1/SLC38A7 | T | 0.82 | 0.09 (0.02) | 1.1x10^-8^ | 0.01 (0.01) | 0.396 |
| rs12421382 |  | 11 | C11orf87 | C | 0.67 | 0.06 (0.01) | 1.7x10^-7^ | 0.00 (0.01) | 0.855 |
| rs12522290 |  | 5 | GRIA1 | C | 0.79 | 0.08 (0.01) | 2.2x10^-8^ | 0.00 (0.02) | 0.768 |
| rs12619354 | rs59979824 (0.90) | 2 | LOC107985969 | T | 0.63 | 0.06 (0.01) | 4.8x10^-7^ | -0.01 (0.01) | 0.454 |
| rs1339227 |  | 6 | RIMS1 | C | 0.65 | 0.06 (0.01) | 6.9x10^-8^ | 0.01 (0.01) | 0.548 |
| rs1501357 |  | 5 | HCN1 | C | 0.21 | 0.08 (0.01) | 1.2x10^-8^ | 0.02 (0.01) | 0.193 |
| rs16867576 |  | 5 | LOC105379072 | A | 0.85 | 0.10 (0.02) | 1.4x10^-8^ | 0.03 (0.02) | 0.129 |
| rs17049247 | rs75575209 (1) | 2 | VRK2 | G | 0.12 | 0.10 (0.02) | 2.9x10^-8^ | 0.02 (0.02) | 0.464 |
| rs17194490 |  | 3 | CNTN4 | T | 0.20 | 0.10 (0.01) | 4.9x10^-11^ | -0.02 (0.02) | 0.308 |
| rs17594665 | rs78322266 (1) | 18 | TCF4 | A | 0.08 | 0.18 (0.03) | 1.1x10^-8^ | 0.01 (0.03) | 0.667 |
| rs1782810 | rs1702294 (1) | 1 | MIR137HG | A | 0.78 | 0.12 (0.01) | 3.1x10^-17^ | 0.01 (0.02) | 0.378 |
| rs2007044 |  | 12 | CACNA1C | G | 0.38 | 0.09 (0.01) | 2.6x10^-17^ | 0.00 (0.01) | 0.696 |
| rs2053079 |  | 19 | ZNF536 | G | 0.26 | 0.07 (0.01) | 3.8x10^-9^ | -0.02 (0.01) | 0.160 |
| rs2068012 |  | 14 | PRKD1 | C | 0.23 | 0.07 (0.01) | 4.1x10^-8^ | 0.03 (0.03) | 0.229 |
| rs2109299 | rs13240464 (1) | 7 | IMMP2L | G | 0.65 | 0.08 (0.01) | 6.0x10^-12^ | -0.01 (0.01) | 0.277 |
| rs211829 |  | 7 | LOC105375451 | T | 0.61 | 0.06 (0.01) | 5.5x10^-7^ | 0.01 (0.01) | 0.313 |
| rs215411 |  | 4 | LOC105374524 | A | 0.34 | 0.06 (0.01) | 1.2x10^-8^ | -0.01 (0.01) | 0.367 |
| rs2239063 |  | 12 | CACNA1C | A | 0.70 | 0.07 (0.01) | 5.4x10^-9^ | 0.01 (0.01) | 0.504 |
| rs2274341 | rs55833108 (0.96) | 10 | NT5C2 | T | 0.25 | 0.07 (0.01) | 9.2x10^-8^ | -0.01 (0.01) | 0.570 |
| rs2514218 |  | 11 | LOC105369501 | C | 0.63 | 0.07 (0.01) | 4.1x10^-10^ | 0.01 (0.01) | 0.536 |
| rs2535627 |  | 3 | ITIH3 / ITIH4 | T | 0.53 | 0.07 (0.01) | 4.0x10^-11^ | 0.00 (0.01) | 0.760 |
| rs2693698 |  | 14 | BCL11B | G | 0.55 | 0.06 (0.01) | 1.4x10^-8^ | 0.03 (0.01) | 0.038 |
| rs2851447 |  | 12 | MPHOSPH9 | G | 0.27 | 0.09 (0.01) | 2.2x10^-14^ | -0.01 (0.01) | 0.328 |
| rs2905425 | rs2905426 (1) | 19 | MAU2 | G | 0.35 | 0.06 (0.01) | 1.0x10^-8^ | 0.00 (0.01) | 0.788 |
| rs3735026 | rs3735025 (1) | 7 | DGKI | T | 0.64 | 0.06 (0.01) | 1.0x10^-8^ | 0.02 (0.02) | 0.266 |
| rs3768641 | rs3768644 (0.92) | 2 | CYP26B1 | C | 0.88 | 0.09 (0.02) | 1.7x10^-5^ | -0.01 (0.02) | 0.608 |
| rs3798149 | rs10043984 (1) | 5 | KDM3B | C | 0.28 | 0.07 (0.01) | 3.3x10^-8^ | 0.01 (0.01) | 0.332 |
| rs3802924 | rs75059851 (1) | 11 | IGSF9B | A | 0.78 | 0.09 (0.01) | 3.5x10^-11^ | 0.02 (0.02) | 0.265 |
| rs3849046 |  | 5 | ETF1 | T | 0.54 | 0.06 (0.01) | 4.8x10^-9^ | 0.01 (0.01) | 0.391 |
| rs4129585 |  | 8 | TSNARE1 | A | 0.44 | 0.08 (0.01) | 2.0x10^-13^ | -0.01 (0.01) | 0.485 |
| rs4240748 |  | 12 | LOC105369900 | G | 0.61 | 0.06 (0.01) | 1.0x10^-7^ | 0.02 (0.01) | 0.217 |
| rs4388249 |  | 5 | MAN2A1 | T | 0.20 | 0.07 (0.01) | 1.0x10^-7^ | -0.05 (0.02) | 0.002 |
| rs4391122 |  | 5 | LOC105378992 | G | 0.48 | 0.08 (0.01) | 1.7x10^-13^ | 0.02 (0.01) | 0.184 |
| rs439639 | rs679087 (1) | 12 | TMTC1 | A | 0.64 | 0.06 (0.01) | 1.1x10^-7^ | 0.00 (0.01) | 0.708 |
| rs4523957 |  | 17 | SMG6 / SRR | T | 0.62 | 0.07 (0.01) | 1.0x10^-9^ | -0.01 (0.01) | 0.510 |
| rs4642619 | rs36068923 (0.94) | 8 | LOC100132280 | A | 0.22 | 0.08 (0.01) | 1.1x10^-10^ | 0.00 (0.01) | 0.920 |
| rs4648845 |  | 1 | PLCH2 | T | 0.46 | 0.07 (0.01) | 4.0x10^-9^ | 0.00 (0.02) | 0.830 |
| rs4664442 | rs2909457 (0.93) | 2 | SLC4A10 | A | 0.45 | 0.06 (0.01) | 5.7x10^-8^ | 0.03 (0.01) | 0.005 |
| rs4702 |  | 15 | FES / FURIN | G | 0.45 | 0.08 (0.01) | 2.3x10^-12^ | 0.02 (0.01) | 0.189 |
| rs4949526 | rs1498232 (1) | 1 | LOC101929406 | T | 0.31 | 0.07 (0.01) | 1.5x10^-9^ | -0.01 (0.01) | 0.543 |
| rs6065094 |  | 20 | PPP1R16B | G | 0.66 | 0.07 (0.01) | 5.5x10^-11^ | -0.01 (0.01) | 0.472 |
| rs6466056 | rs6466055 (0.93) | 7 | SRPK2 | T | 0.36 | 0.07 (0.01) | 2.9x10^-9^ | 0.00 (0.01) | 0.848 |
| rs6471814 | rs6984242 (0.96) | 8 | LOC107986889 | T | 0.42 | 0.06 (0.01) | 2.4x10^-9^ | -0.01 (0.01) | 0.622 |
| rs6670165 |  | 1 | BRINP2 | T | 0.22 | 0.07 (0.01) | 1.2x10^-7^ | -0.01 (0.01) | 0.553 |
| rs6704641 |  | 2 | SATB2 | A | 0.78 | 0.076202 | 3.4x10^-8^ | 0.02 (0.02) | 0.264 |
| rs6704768 |  | 2 | GIGYF2 | G | 0.45 | 0.073797 | 3.1x10^-12^ | 0.00 (0.01) | 0.835 |
| rs715170 |  | 18 | LINC01539 | C | 0.71 | 0.066204 | 3.5x10^-8^ | -0.03 (0.01) | 0.020 |
| rs7267348 |  | 20 | PTGIS | C | 0.27 | 0.064197 | 1.2x10^-7^ | -0.01 (0.01) | 0.713 |
| rs7403809 | rs7405404 (1) | 16 | SHISA9 | T | 0.26 | 0.08 (0.01) | 4.6x10^-10^ | 0.03 (0.01) | 0.036 |
| rs7432375 |  | 3 | STAG1 | G | 0.58 | 0.07 (0.01) | 5.3x10^-11^ | -0.01 (0.01) | 0.408 |
| rs7801375 |  | 7 | LOC107986849 | G | 0.82 | 0.08 (0.01) | 2.3x10^-8^ | 0.02 (0.02) | 0.222 |
| rs7893279 |  | 10 | CACNB2 | T | 0.85 | 0.11 (0.02) | 3.6x10^-11^ | -0.01 (0.02) | >0.999 |
| rs7927176 | rs77502336 (0.96) | 11 | GRAMD1B | G | 0.32 | 0.06 (0.01) | 1.4x10^-8^ | -0.01 (0.02) | 0.522 |
| rs8042374 |  | 15 | CHRNA3 | A | 0.75 | 0.09 (0.01) | 1.9x10^-12^ | 0.00 (0.01) | 0.802 |
| rs8044995 |  | 16 | NFATC3 | A | 0.19 | 0.08 (0.01) | 3.3x10^-8^ | -0.01 (0.02) | 0.577 |
| rs832187 |  | 3 | C3orf49 / THOC7 | C | 0.38 | 0.06 (0.01) | 2.6x10^-8^ | -0.02 (0.01) | 0.170 |
| rs9420 |  | 11 | C11orf31 / TMX2-CTNND1 | A | 0.33 | 0.06 (0.01) | 6.7x10^-8^ | 0.01 (0.01) | 0.445 |
| rs950169 |  | 15 | ADAMTSL3 | C | 0.71 | 0.08 (0.01) | 7.6x10^-11^ | 0.01 (0.01) | 0.308 |
| rs9636107 |  | 18 | TCF4 | G | 0.48 | 0.08 (0.01) | 9.1x10^-13^ | 0.04 (0.01) | 6.2x10^-4^ |
| rs9841616 |  | 3 | SOX2-OT | T | 0.80 | 0.08 (0.01) | 1.7x10^-8^ | -0.05 (0.02) | 0.004 |
| rs9922678 |  | 16 | GRIN2A | A | 0.31 | 0.07 (0.01) | 6.7x10^-9^ | -0.02 (0.01) | 0.233 |
| rs10504857 | rs7819570 (1) | 8 | LOC105375629 | G | 0.21 | 0.07 (0.01) | 4.4x10^-8^ | 0.01 (0.02) | 0.549 |
| rs10779702 | chr1_8424984_Db (0.97) | 1 | RERE | A | 0.35 | 0.06 (0.01) | 1.3x10^-8^ | 0.00 (0.01) | 0.886 |
| rs10927075 | chr1_243881945_I (1) | 1 | AKT3 | T | 0.66 | 0.05 (0.01) | 1.3x10^-6^ | 0.03 (0.01) | 0.039 |
| rs11167597 | rs111294930 (0.97) | 5 | LINC01470 | T | 0.70 | 0.07 (0.01) | 2.1x10^-8^ | 0.01 (0.01) | 0.350 |
| rs11683083 | chr2_146436222_I (1) | 2 | LOC105373664 | G | 0.19 | 0.08 (0.01) | 1.6x10^-8^ | -0.02 (0.02) | 0.270 |
| rs12063329 | rs140505938 (1) | 1 | LOC105371431 | T | 0.81 | 0.09 (0.01) | 2.6x10^-9^ | 0.00 (0.02) | 0.973 |
| rs12659129 | chr5_140143664_I (1) | 5 | LOC100421074 | T | 0.47 | 0.05 (0.01) | 9.6x10^-7^ | -0.02 (0.01) | 0.180 |
| rs12716972 | rs12691307 (0.98) | 16 | KCTD13 | A | 0.47 | 0.06 (0.01) | 3.6x10^-9^ | 0.00 (0.01) | 0.923 |
| rs13074054 | chr3_180594593_I (0.99) | 3 | LOC105374240 | A | 0.76 | 0.08 (0.01) | 8.8x10^-10^ | 0.00 (0.01) | 0.902 |
| rs13230421 | rs12704290 (0.96) | 7 | GRM3 / LOC105375382 | T | 0.85 | 0.10 (0.02) | 2.8x10^-10^ | 0.00 (0.02) | 0.802 |
| rs17149781 | chr7_24747494_D (0.91) | 7 | MPP6 | G | 0.14 | 0.08 (0.02) | 1.6x10^-6^ | 0.00 (0.02) | 0.856 |
| rs2927177 | rs2973155 (0.98) | 5 | none | T | 0.64 | 0.06 (0.01) | 5.3x10^-9^ | -0.01 (0.01) | 0.452 |
| rs2955358 | rs8082590 (1) | 17 | GID4 | T | 0.34 | 0.06 (0.01) | 1.6x10^-8^ | 0.00 (0.01) | 0.803 |
| rs4128242 | chr18_52749216_D (1) | 18 | LOC105372125 | T | 0.58 | 0.07 (0.01) | 5.5x10^-11^ | 0.00 (0.01) | 0.846 |
| rs4522708 |  | 22 | CACNA1I | A | 0.39 | 0.07 (0.01) | 2.4x10^-10^ | -0.02 (0.01) | 0.134 |
| rs6461049 | chr7_2025096_I (0.93) | 7 | MAD1L1 | T | 0.56 | 0.08 (0.01) | 3.7x10^-13^ | -0.01 (0.02) | 0.737 |
| rs7096169 | rs11167597 (0.99) | 10 | BORCS7-ASMT / BORCS7 | A | 0.64 | 0.09 (0.01) | 4.8x10^-17^ | -0.04 (0.01) | 5.1x10^-4^ |
| rs7140568 | rs12887734 (1) | 14 | APOPT1 | T | 0.30 | 0.08 (0.01) | 5.7x10^-13^ | 0.00 (0.01) | 0.777 |
| rs7588380 | rs11685299 (1) | 2 | CUL3 | T | 0.65 | 0.06 (0.01) | 7.3x10^-8^ | 0.02 (0.01) | 0.089 |
| rs788017 | rs6434928 (1) | 2 | SF3B1 | T | 0.34 | 0.07 (0.01) | 2.4x10^-9^ | 0.01 (0.01) | 0.434 |
| rs9876421 | rs75968099 (0.93) | 3 | LOC105377031 | T | 0.36 | 0.08 (0.01) | 7.2x10^-12^ | 0.00 (0.01) | 0.852 |

**Table S2. Correlation matrix of SNPs that predict smoking initiation (r^2^**)

|  | **rs6265** | **rs4923460** | **rs1304100** | **rs6484320** |
| --- | --- | --- | --- | --- |
| rs6265 | 1 | 0.817 | 0.652 | 0.603 |
| rs4923460 | 0.817 | 1 | 0.798 | 0.775 |
| rs1304100 | 0.652 | 0.798 | 1 | 0.598 |
| rs6484320 | 0.603 | 0.775 | 0.598 | 1 |

**Table S3. SNPs associated with smoking initiation p<10^-6^**

| **SNP name** | **Chrom-osome** | **Nearest gene (within 100k bp)** | **Reference allele** | **Freq ref allele** | **Gene-smoking initiation estimate (se)** | **p-value** | **Gene-schizophrenia estimate (se)** | **p-value** |
| --- | --- | --- | --- | --- | --- | --- | --- | --- |
| rs926246 | 1 | LOC100420339 | T | 0.84 | 0.09 (0.02) | 8.61x10^-6^ | 0.02 (0.02) | 0.215 |
| rs7548367 | 1 | LOC102723924 | C | 0.66 | 0.06 (0.01) | 9x10^-6^ | -0.01 (0.01) | 0.414 |
| rs11892348 | 2 | KCNS3 | A | 0.35 | -0.05 (0.01) | 8.61x10^-6^ | -0.02 (0.01) | 0.114 |
| rs10937751 | 4 | MRFAP1L1 | A | 0.30 | 0.07 (0.01) | 2.84x10^-6^ | 0.01 (0.01) | 0.244 |
| rs7663808 | 4 | LOC105374492 | A | 0.59 | -0.06 (0.01) | 8.11x10^-6^ | -0.01 (0.01) | 0.238 |
| rs10013579 | 4 | none | T | 0.63 | 0.06 (0.01) | 3.54x10^-6^ | 0.01 (0.01) | 0.368 |
| rs1448438 | 4 | LOC105377509 | T | 0.72 | 0.06 (0.01) | 6.05x10^-6^ | -0.01 (0.01) | 0.330 |
| rs13131292 | 4 | LOC107986241 | A | 0.19 | -0.11 (0.02) | 7.57x10^-6^ | 0.03 (0.02) | 0.106 |
| rs725695 | 5 | TENM2 | A | 0.41 | -0.05 (0.01) | 4.95x10^-6^ | 0.00 (0.01) | 0.927 |
| rs1986692 | 7 | EXOC4 | A | 0.60 | 0.06 (0.01) | 7.37x10^-6^ | 0.03 (0.01) | 0.018 |
| rs2449222 | 8 | CSMD1 | T | 0.88 | -0.09 (0.02) | 5.30x10^-6^ | 0.01 (0.02) | 0.728 |
| rs10108954 | 8 | MFHAS1 | T | 0.07 | -0.17 (0.03) | 1.55x10^-6^ | 0.06 (0.04) | 0.072 |
| rs16904189 | 8 | FAM49B | T | 0.92 | -0.15 (0.03) | 7.46x10^-6^ | -0.01 (0.03) | 0.834 |
| rs6265 | 11 | BDNF | T | 0.21 | 0.063 (0.015) | 7.72x10^-8^ | -0.05 (0.01) | 7.96x10^-5^ |
| rs11030084 | 11 | BDNF-AS/LINC00678 | T | 0.21 | -0.07 (0.01) | 7.18x10^-6^ | -0.05 (0.01) | 1.41x10^-4^ |
| rs1817648 | 12 | ALG10B | T | 0.47 | -0.05 (0.01) | 7.84x10^-6^ | 0.00 (0.01) | 0.900 |
| rs739484 | 12 | CUX2 | T | 0.14 | -0.09 (0.02) | 9.00x10^-6^ | 0.00 (0.02) | 0.938 |
| rs11067275 | 12 | LOC107984437 | T | 0.67 | 0.07 (0.01) | 2.70x10^-6^ | 0.01 (0.01) | 0.394 |
| rs11246771 | 12 | SFSWAP | T | 0.17 | -0.08 (0.02) | 4.91x10^-6^ | 0.03 (0.02) | 0.046 |
| rs9521281 | 13 | MYO16 | T | 0.20 | -0.07 (0.02) | 8.42x10^-6^ | 0.01 (0.01) | 0.458 |
| rs241526 | 14 | LOC105370512 | T | 0.48 | -0.05 (0.01) | 8.55x10^-6^ | 0.00 (0.01) | 0.857 |
| rs11570441 | 17 | CDC27 | C | 0.11 | 0.11 (0.02) | 4.24x10^-6^ | -0.01 (0.02) | 0.487 |
